# Efficacy of acupuncture as an adjunctive treatment to patients with stable COPD: a multicenter, randomized, sham-controlled trial protocol

**DOI:** 10.1186/s12906-024-04412-6

**Published:** 2024-03-07

**Authors:** Liuyang Huang, Sha Yang, Guixing Xu, Qin Luo, Chunyan Yang, Hao Tian, Yilin Liu, Zhuo Zhou, Fengyuan Huang, Siyao Gong, Qian Li, Xin Yu, Ming Chen, Dan Huang, Yunyu Liu, Juan Tang, Ruixin Zhang, Xin Sun, Guangbing Lu, Chunfang Zeng, Shuangchun Ai, Bin Li, Jian qin Chen, Quan Luo, Chan Xiong, Zhi Zou, Qiang Hu, Xiaochao Luo, Ling Li, Mingsheng Sun, Fang Zeng, Fanrong Liang

**Affiliations:** 1 Acupuncture and Tuina School, Chengdu University of Traditional Chinese Medicine, No. 37, Shi’er Qiao Road, Jinniu District, Chengdu, 610075 Sichuan China; 2 School of Health and Wellness, Panzhihua University, No. 10, North Section of Sanxian Avenue, East District, Panzhihua, 617000 Sichuan China; 3 Department of Acupuncture and Tuina Medicine, Guangyuan Hospital of Traditional Chinese Medicine, No.133 Jianshe Road, Lizhou District, Guangyuan, 628099 Sichuan China; 4 Chinese Evidence-Based Medicine Center and Cochrane China Center, West China Hospital, Sichuan University, Chengdu, 610041 Sichuan China; 5 Department of Respiratory Medicine, Chinese Traditional Medicine Hospital of Meishan, No. 9, North Section of Mindong Avenue, Dongpo District, Meishan, 620010 Sichuan China; 6 Department of Respiratory Medicine, Deyang People’s Hospital, No.173, Section 1, Taishan North Road, Jingyang District, Deyang, 618009 Sichuan China; 7 Department of Acupuncture and Tuina Medicine, Mianyang Hospital of TCM, No.14, Fucheng Road, Fucheng District, Mianyan, 621053 Sichuan China; 8 Department of Respiratory Medicine, Guangyuan Hospital of Traditional Chinese Medicine, No.133 Jianshe Road, Lizhou District, Guangyuan, 628099 Sichuan China; 9 Department of Respiratory Medicine, Hospital of Chengdu University of TCM, No. 39, Shi’er Qiao Road, Jinniu District, Chengdu, 610075 Sichuan China; 10 Department of Traditional Chinese Medicine, Chengdu Second People’s Hospital, No.10 Qingyun South Street, Jinjiang District, Chengdu, 610021 Sichuan China; 11 Department of Respiratory Medicine, Chengdu TCM Hospital of Pidu District, No.169, Sec.1, Zhongxin Avenue, Pidu District, Chengdu, 611730 Sichuan China; 12 Department of Respiratory Medicine, Meishan People’s Hospital, No. 288, South Section 4Dongpo Avenue, Dongpo District, Meishan, 620020 Sichuan China; 13 Department of Respiratory Medicine, Panzhihua Integrated TCM and Western Medicine Hospital, No. 27, Taoyuan Street, Bingcaogang, East District, Panzhihua, 617099 Sichuan China

**Keywords:** Acupuncture, COPD, Randomized controlled trial, Protocol

## Abstract

**Background:**

Chronic obstructive pulmonary disease (COPD) is a common respiratory disease and the third leading cause of death worldwide. Previous evidence has shown that acupuncture may be an effective complementary alternative therapy for stable COPD. However, large-sample, rigorously designed long-term follow-up studies still need to be completed. Notably, the relationship between the frequency of acupuncture and clinical efficacy in studies on acupuncture for stable COPD still needs further validation. This study aims to evaluate the efficacy and safety of acupuncture for stable COPD and further investigate the dose–effect relationship of acupuncture.

**Methods/design:**

This is a multicenter, randomized, controlled trial that uses central randomization to randomly allocate 550 participants in a 1:1:1:1:1 ratio to once a week acupuncture group, twice a week acupuncture group, three times a week acupuncture group, sham acupuncture group and waiting-list control group. The sham acupuncture group will receive placebo acupuncture treatments three times per week, and the waiting-list control group will not receive any form of acupuncture intervention. The study consists of a 2-week baseline, 12-week of treatment, and 52-week of follow-up. Patients with COPD between 40 to 80 years old who have received stable Western medication within the previous 3 months and have had at least 1 moderate or severe acute exacerbation within the past 1 year will be included in the study. Basic treatment will remain the same for all participants. The primary outcome is the proportion of responders at week 12. Secondary outcomes include the proportion of responders at week 64, change in the St. George's Respiratory Questionnaire (SGRQ) Scale, change in the Modified-Medical Research Council (mMRC) Scale, change in the COPD Assessment Test (CAT) Scale, change in the Lung Function Screening Indicators (LFSI), change in the 6-min walk distance (6-MWD), change in Short-Form 36 Health Survey (SF-36) Scale, the number of moderate and severe acute exacerbations and adverse event rate during the follow-up period.

**Discussion:**

This study will provide robust evidence on whether acupuncture is safe and effective for treating stable COPD. Meanwhile, comparing the differences in efficacy between different acupuncture frequencies will further promote the optimization of acupuncture for stable COPD.

**Trial registration:**

This study was registered in the Chinese Clinical Trial Registry (ChiCTR2200058757), on April 16, 2022.

**Supplementary Information:**

The online version contains supplementary material available at 10.1186/s12906-024-04412-6.

## Introduction

Chronic obstructive pulmonary disease (COPD), characterized by airflow limitation and persistent respiratory symptoms caused by a combination of small airway disease (obstructive bronchitis) and lung parenchymal destruction (emphysema), is the third most common cause of death [1, 2]. In 2019, 212.3 million prevalent cases of COPD were reported globally, including 3.3 million deaths and 74.4 million disability-adjusted life years [3, 4]. Continued exposure to COPD risk factors and an aging population have made COPD a global public health challenge [5]_._ Clinical treatment of stable COPD aims to reduce symptoms, decrease the frequency and severity of acute exacerbations, improve health status, and enhance exercise tolerance. Despite the importance of bronchodilators, glucocorticoids, mucolytics, and antibiotics as clinically indicated drugs in treating stable COPD, there are inevitable side effects [6]. For example, beta_2_-agonists may cause hypokalemia and tardive dyskinesia [7, 8], antimuscarinic drugs cause dry mouth [9], glucocorticoids increase the incidence of pneumonia [10], and antibiotics cause bacterial resistance and tinnitus [11]. Moreover, some patients still have symptoms that cannot be effectively controlled based on conventional medications. Therefore, it is imperative to explore new treatment options that are safe and effective, with fewer side effects, to improve patient symptoms further, reduce acute exacerbations, and minimize the side effects of conventional drug therapy.

Acupuncture has been widely used worldwide as an essential complementary alternative therapy for preventing and treating many chronic diseases. Previous studies have shown that the Borg scale after the 6-min walk test (6-MWT) was significantly better in the acupuncture group than in the control group (treatment as usual). That 6-min walk distance (6-MWD), oxygen saturation, and lung function also improved compared to the control group [12, 13]. Compared with the placebo acupuncture group, the Borg scale score, 6-MWD, pulmonary ventilation function, and St George's Respiratory Questionnaire (SGRQ) were significantly improved in the acupuncture group, and the body mass index (BMI), airflow obstruction, dyspnea, and exercise capacity (BODE) index also experienced improvement [14–17]. The above studies have shown that acupuncture helps to reduce dyspnea, improve exercise capacity, and improve lung function and nutritional status in patients with stable COPD. Systematic reviews based on evidence-based medicine have also demonstrated acupuncture as an effective adjunctive non-pharmacological treatment to improve the quality of life and dyspnea in COPD medically treated patients. However, there still needs to be high-quality randomized controlled trials to validate it [18, 19].

Acupuncture dose affects the acupuncture effect and determines the treatment and prognosis of the disease [20]. Among these, the frequency of acupuncture is essential in influencing the outcome and determining the cost of medical care. However, the relationship between acupuncture frequency and efficacy remains largely unexplored. A recent systematic review found that 36 acupuncture treatments had the best clinical response in patients with major depression compared to 24 and 12 treatments [21]. Previous clinical studies on acupuncture for stable COPD had a treatment frequency of 1–3 times per week, with a treatment period of 2–12 weeks and a total number of sessions ranging from 6–24 [12, 14–17, 22–24]. No clinical studies comparing different frequencies of acupuncture for stable COPD are available; thus, it is not possible to determine the preferred frequency and intervention period for acupuncture for COPD.

Therefore, this study designed a multi-centered, large-sample randomized controlled trial with the aim of (1) evaluate the efficacy and safety of acupuncture for stable COPD and (2) exploring the frequency of acupuncture suitable for stable COPD.

## Methods

### Study design

This study outlines a protocol for a prospective, multicenter, randomized controlled trial in which participants will be assigned to 5 parallel groups. The protocol reporting follows the SPIRIT statement [25], and future trials will follow the Consolidated Standards of Reporting Trials (CONSORT) guidelines [26], including an extension of the Revised Standards for Reporting Interventions in Clinical Trials of Acupuncture (STRICTA) [27].

**Fig. 1:**
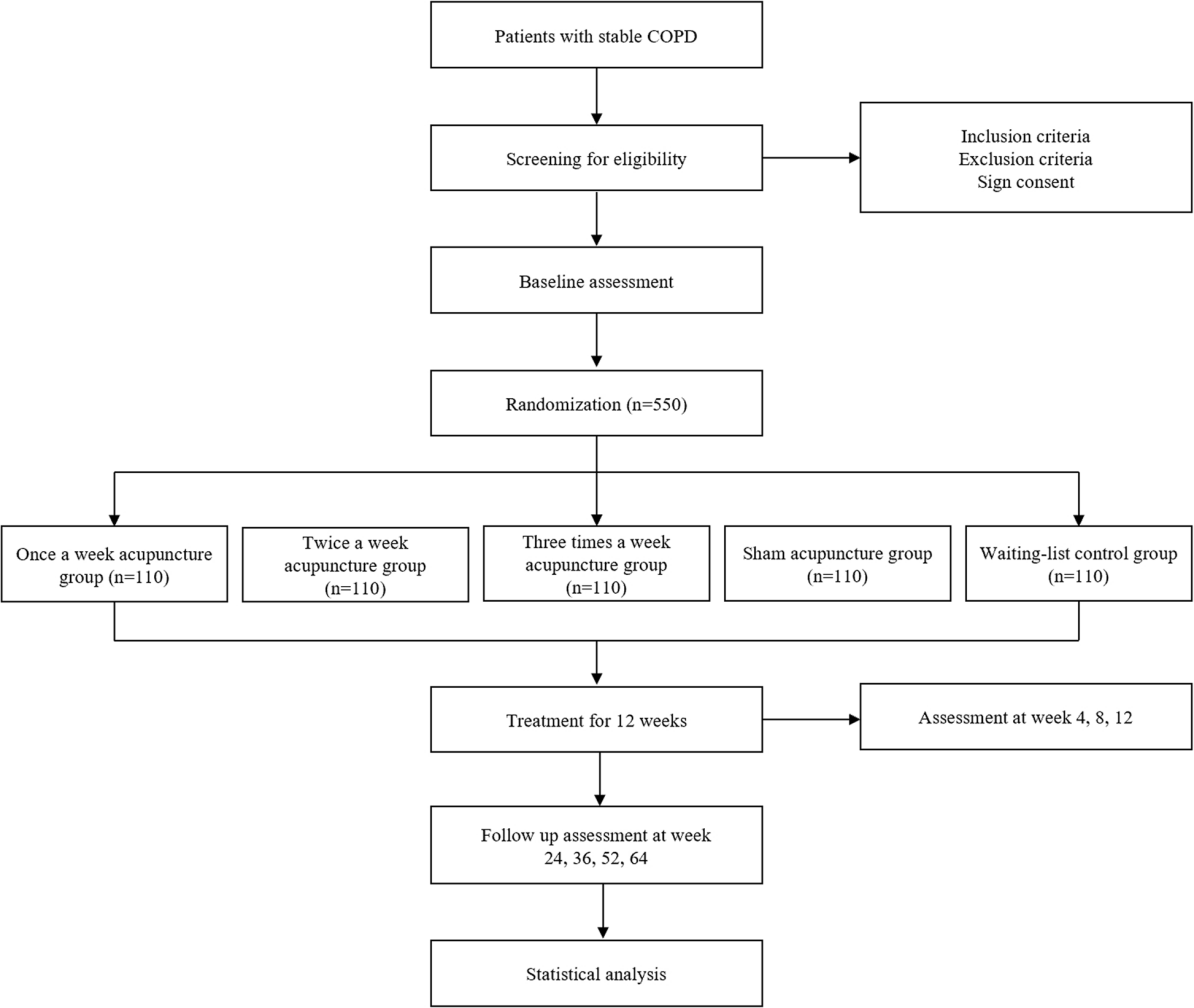
Research flow chart

550 participants with stable COPD who meet the diagnostic criteria will be enrolled in this study. All participants are coming from 11 tertiary hospitals in Sichuan Province, China: Hospital of Chengdu University of TCM, Sichuan Provincial People's Hospital, Chengdu First People's Hospital, Chengdu Second People's Hospital, Chinese Traditional Medicine Hospital of Meishan, Mianyang Hospital of Traditional Chinese Medicine, Deyang People's Hospital, Guangyuan Hospital of Traditional Chinese Medicine, Panzhihua Integrated TCM and Western Medicine Hospital, Meishan People's Hospital, and Chengdu TCM Hospital of Pidu District. The entire study period consists of 2 weeks of baseline, 12 weeks of treatment, and 52 weeks of follow-up. A flow chart of the trial design is shown in Fig. 1, and patient enrollment, intervention, and assessment times are shown in Table 1.

**Table 1:**
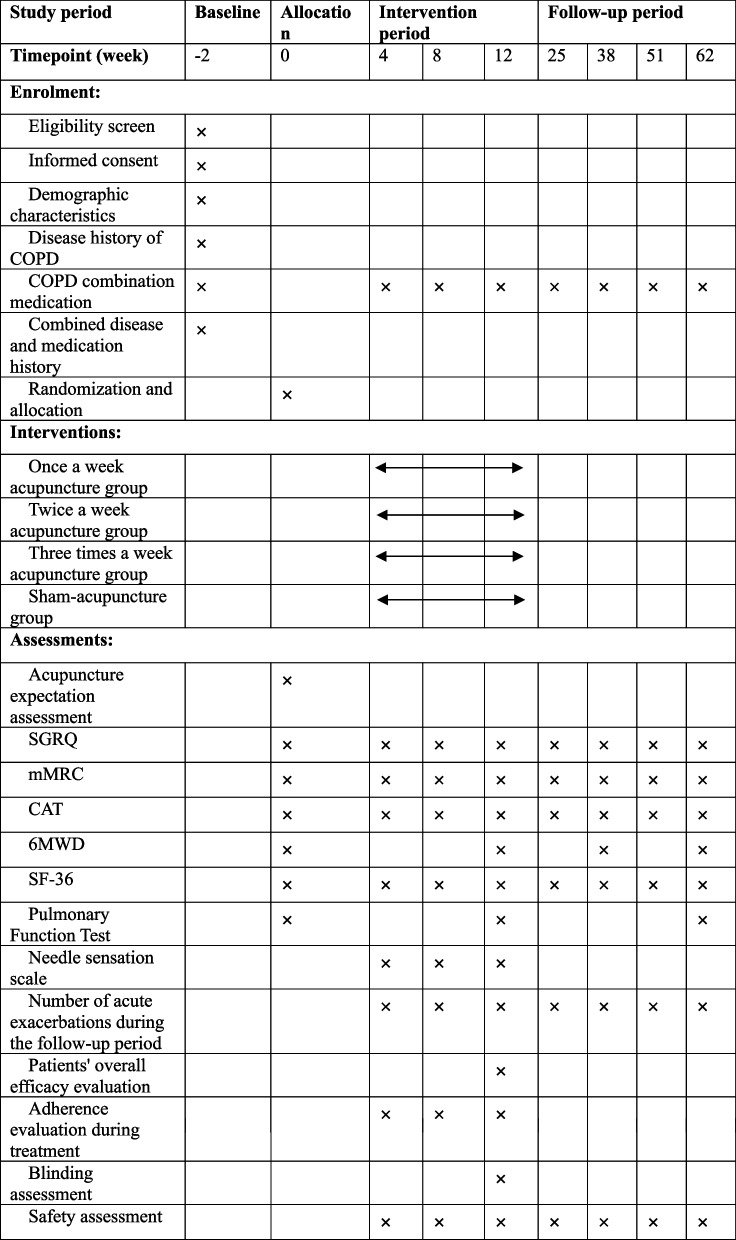
Timepoint for recruitment, interventions and evaluation

*SGRQ *St. George's Respiratory Questionnaire, *mMRC *Modified-Medical Research Council, *CAT *COPD Assessment Test, *6-MWD *6-min walk distance, *SF-36 *Short-Form 36 Health Survey

### Randomization and blinding

The central randomization system is used to conduct dynamic block group randomization stratified by center, and participants are randomly assigned in a ratio of 1:1:1:1:1 to once a week acupuncture group, twice a week acupuncture group, three times a week acupuncture group, sham-acupuncture group, and waiting-list control group. Beijing Bioknow Information Technology Co. will perform randomization. An independent researcher from each center not involved in treatment and outcome assessment is responsible for logging on to the central randomization system and entering randomization information (including the center number and the participant's name in *pinyin* initials). Randomization numbers and group assignments will be emailed to this independent assessor. Patients will be settled in separate treatment rooms to avoid communication with each other. Evaluators, statisticians, and participants will be blind to treatment allocations. The location of acupoints and the use of assistive devices will be the same for the acupuncture and sham acupuncture groups.

### Participants

#### Inclusion criteria

Participants who meet the diagnostic criteria for COPD according to the Global Initiative for COPD and conform to all the following criteria will be included in the study [28]: 1) stable condition without acute exacerbation in the last 4 weeks; 2) age between 40 and 80 years, both sexes; 3) FEV1pre between 25 and 80% after inhaled bronchodilators; 4) at least 1 moderate or severe acute exacerbation within the past year; 5) receiving stable Western medication within the previous 3 months; 6) willing to cooperate with the study and sign an informed consent form.

#### Exclusion criteria

Patients who meet any of the following conditions will be excluded: 1) combination of the severe cardiovascular, neurological, hematological, immune system, and malignant tumor diseases; 2) combined respiratory diseases that significantly impact the study (active tuberculosis, bronchial asthma, severe bronchiectasis, primary pulmonary hypertension, interstitial lung disease); 3) history of segmentectomy, wedge resection, lobectomy, total pneumonectomy, or lung volume reduction (including bronchoscopic lung volume reduction) for COPD; 4) continuous oxygen therapy is required according to long-term oxygen therapy (LTOT) criteria (oxygen therapy duration > 15 h/d) [29]; 5) unable to perform pulmonary function tests, unable to walk independently, unable to cooperate in completing questionnaires; 6) persistent breakage or infection of skin tissue at the needle site, coagulation dysfunction, skin allergy; 7) participated in another clinical trial or performed acupuncture for respiratory disease within the last 3 months; 8) pregnant and lactating women.

### Patient recruitment

Recruitment strategies include poster recruitment, community recruitment, online recruitment, respiratory outpatients, and previous inpatients. Patients will be informed of study specifics, including study purpose, subgroup status, interventions, treatment period, benefits, and potential risks. A respiratory specialist will diagnose patients who agree to participate in this study and must sign an informed consent form before randomization to the group. All patient information will be kept confidential. Patients may withdraw at any trial stage, and the reasons for patient withdrawal will be well documented.

### Intervention and comparison

All patients will be given interventions based on conventional treatment. During the baseline period, all medications used by the subject to treat COPD and other underlying conditions will be recorded in detail on a case report form (CRF), including the name of the medication, the dose used, the frequency of use, the route of administration, and the start and end times. Any changes in the subject's medication throughout the study will also be recorded in detail. Medications used by patients to treat COPD will be categorized based on GOLD guideline criteria. A respiratory medicine specialist will assess the possibility of continuing acupuncture treatment during acute exacerbations. If a patient is hospitalized for an acute exacerbation, treatment will be continued 1 week after discharge, and the number of treatments delayed due to hospitalization will be replenished. During the study, we will discourage patients from using medications outside the guidelines to treat COPD, including herbal ones. However, if the patient has already used them, they must be documented accordingly.

The classification of basic therapeutic drugs is based on the 2023 GOLD guideline criteria: beta2 agonists (including fenoterol, levosalbutamol, formoterol, salmetero), anticholinergics (including ipratropium bromide, adiponium bromide), and short-acting beta2 agonists and anticholinergics in combination with inhalation devices (including fenoterol/ipratropium bromide, salbutamol/ipratropium bromide), long-acting beta2 agonists and anticholinergic combination inhalation devices (including formoterol/adiponium bromide, formoterol/glumonium bromid), methylxanthines (including aminophylline, theophylline), long-acting beta2 agonists in combination with hormonal inhalation devices (including formoterol/beclomethasone, formoterol/budesonid), and phosphodiesterase-4 inhibitors (including roflumilast).

**Fig. 2:**
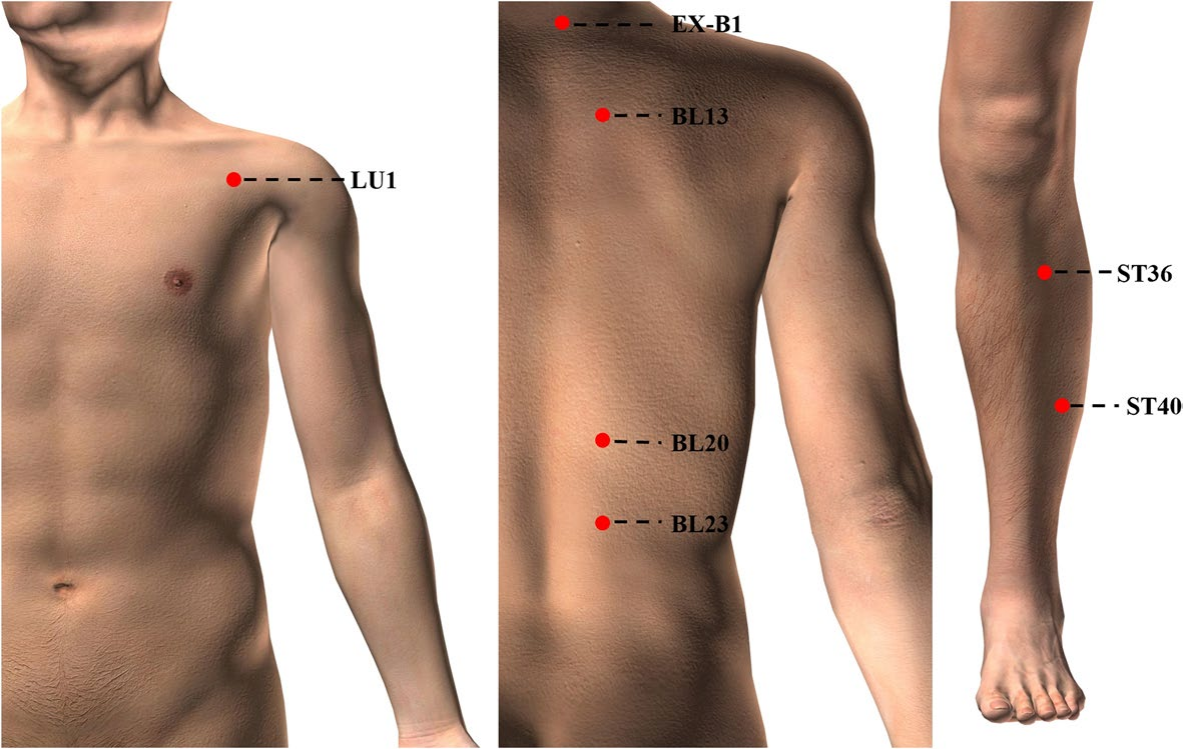
Locations of acupoints

The treatment protocol was developed based on the preliminary literature collation and a consensus meeting of acupuncture experts. 7 pairs of commonly used and effective acupoints for the treatment of COPD were selected, including bilateral Zhongfu (LU1), Dingchuan (EX-B1), Feishu (BL13), Pishu (BL20), Shenshu (BL23), Zusanli (ST36), and Fenglong (ST40). The acupuncture group and the sham acupuncture group used the same acupoints. Details of each acupoint are shown in Table 2 and Fig. 2. The acupuncturists are licensed and have at least 3 years of clinical experience. All acupuncturists will receive training on acupoint positioning and manipulation before the trial. Each sub-center is assigned an acupuncturist responsible for treating all patients within that center. The acupuncture and sham-acupuncture groups will use a rubber base on the acupoints and perform needling (lifting, thrusting, twisting and rotating manipulation) at 10-min intervals to achieve a better blinding effect.

**Table 2:** Locations of acupoints

Acupoints	Locations
Zhongfu (LU1)	On the upper lateral chest, 6 cun from the middle of the chest, level in the first intercostal space
Dingchuan (EX-B1)	0.5 cun lateral to DU14
Feishu (BL13)	1.5 cun lateral to the depression below the spinous process of the 3th thoracic vertebra
Pishu (BL20)	1.5 cun lateral to the depression below the spinous process of the 11th thoracic vertebra
Shenshu (BL23)	1.5 cun lateral to the depression below the spinous process of the 2th lumbar vertebra
Zusanli (ST36)	3 cun directly below ST35, and one finger-breadth lateral to the anterior border of the tibia
Fenglong (ST40)	One finger-breadth lateral to ST38, and at the midpoint of the line joining ST35 and the tip of the external malleolus

### Manual acupuncture (MA) group

There are 3 acupuncture groups, which include once a week acupuncture group, twice a week acupuncture group and three times a week acupuncture group. The patient remains prone during acupuncture, and a soft pillow is placed on the front of the patient's chest to elevate the front of the chest to facilitate needling. The acupuncturist will sterilize the skin around the acupoint using 75% alcohol, then attach a rubber base to the acupoint and insert the needle through the rubber base into the acupoint. For LU1, a 1 cun needle (0.25 × 25 mm, Suzhou Hua Tuo, China) is used, and the needle is inserted in a lateral direction for approximately 15–20 mm. The needle is angled at 45° toward the spine for the dorsal acupoints. BL13 is inserted with a 0.5 cun needle (0.25 × 13 mm, Suzhou Hua Tuo, China) for approximately 5–10 mm. EX-B1 and BL20 are inserted with a 1 cun needle (0.25 × 25 mm, Suzhou Hua Tuo, China) for approximately 15–20 mm. BL23, ST36, and ST40, using a 1.5-inch needle (0.25 × 40 mm, Suzhou Huatuo, China), are inserted for approximately 25- 30 mm. After piercing the needle, gentle lifting, thrusting, swirling, and rotating movements are performed on the needle body to achieve *deqi* sensation (soreness, numbness, distension, or heaviness) [30]. The patient's degree of *deqi* sensation will be assessed immediately after the patient has *deqi* sensation [31]. The needles will be retained for 30 min each time, and *deqi* sensation operation will be performed every 10 min, about 15 s each time. When removing the needle, the acupuncturist uses a dry, sterilized cotton ball to gently press the needled area to avoid bleeding.

Once a week acupuncture group: needling once a week with 6-day intervals, 12 times for 12 weeks. Twice a week acupuncture group: 2 needling sessions per week, with a 3-day interval between needling sessions, for 24 needling sessions over 12 weeks. Three times a week acupuncture group: 3 times a week with 1-day intervals for 36 needling sessions over 12 weeks.

### Sham acupuncture (SA) group

**Fig. 3:**
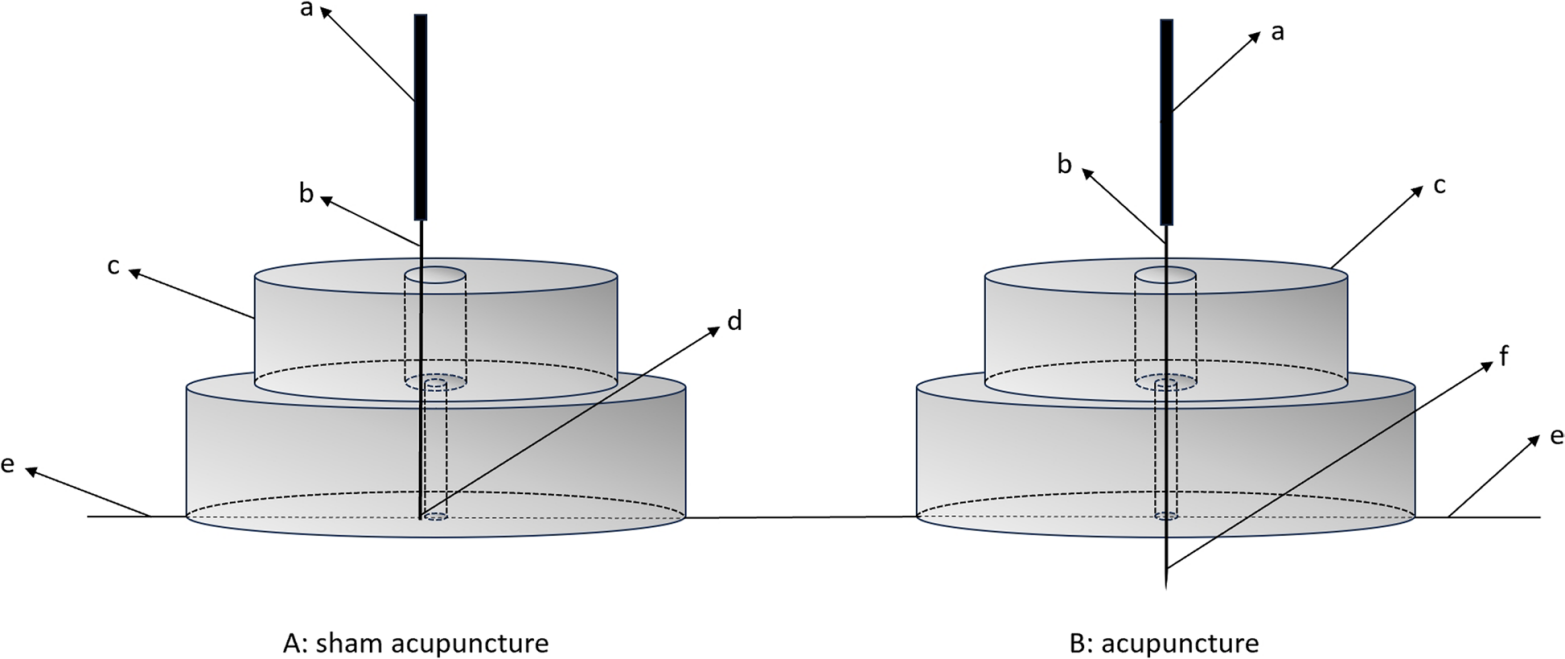
Illustration of acupuncture. a. Needle handle; b. Needle body; c. Plastic cover; d. Blunt tip of the placebo needle; e. Skin; f. Needle tip

The SA group will receive placebo acupuncture in bilateral LU1、EX-B1、BL13、BL20、BL23、ST3, and ST40 on a treatment protocol similar to the acupuncture group. The frequency of the SA treatment is three times a week, with a one-day interval between each treatment, for a total of 36 treatments over 12 weeks. After routine skin disinfection, a rubber soft pad is adhered to the acupoint. A disposable blunt needle (0.3 × 0.13 mm) is used to pierce the base of the rubber soft pad so that the needle body is fixed vertically on the patient's skin. The patient will feel pain but without puncturing the skin. The treatment is simulated by twisting, turning, lifting, and inserting manipulation every 10 min during the treatment, gently swinging the needle body, but without producing a *deqi* sensation. The needles are left for 30 min. Considering the ethical requirements, a free compensatory acupuncture treatment will be given at the end of the trial (Fig. 3).

### Waiting-list control group

No change in the original primary treatment of COPD patients and no acupuncture intervention will be given. Patients will be required to complete the appropriate tests and scale assessments at the appropriate time points and be given free acupuncture treatment at the end of the trial.

### Blinding assessment

To verify the success of blinding, participants will be asked at the end of the 12-week treatment to answer the question: “do you think you received a traditional acupuncture treatment? (Yes or no).

### Outcome measurements

The outcome measurements and the evaluation times for these measurements are listed in Table 1.

#### Primary outcome

The primary outcome is the proportion of responders at week 12. Responder is defined as participants with a ≥ 4-unit decline in total SGRQ score compared with baseline. A decline of at least 4 points in the total SGRQ score has been determined to be the minimal clinically important difference (MCID) [32]. ≥ 4-unit worsening of SGRQ total score was associated with increased risk of death. The SGRQ is one of the most widely used scales for measuring health impairment and quality of life in adult patients with respiratory diseases, including 1. symptoms (frequency and severity of symptoms); 2. activity (ability to cause breathlessness or limitation of activity due to breathlessness); and 3. impact on daily activities (impairment of social competence and psychological impairment due to airway disease). The above 3 main aspects are scored according to the weights of different questions to obtain the symptom score, activity score and impact score, and finally, the total score is summarized. Higher patient scores indicate poorer quality of life [33]. SGRQ scores will be evaluated at weeks 0, 4, 8, and 12 of enrollment and every 3 months during the follow-up period.

#### Secondary outcomes

Secondary outcomes included the following eight items: 1) the proportion of responders at week 64; 2) change in the SGRQ Scale; 3) change in the mMRC Scale; 4) change in the CAT Scale; 5) change in the Lung Function Screening Indicators (including FEV1, FEV1%pre, FVC and FEV1/FVC); 6) change in the 6MWD;7) change in SF-36 Scale; 8) the number of moderate and severe acute exacerbations during the follow-up period.

The mMRC consists of five grades (0 to 4) and contains statements that describe a range of physical limitations associated with dyspnea. It measures the degree to which a person has dyspnea during activities (such as "strenuous exercise") or limits a person's behavior (such as "too breathless to leave the house") [34]. The CAT consists of 8 items that involve breathing (cough, phlegm, chest tightness, and dyspnea) and other symptoms (low energy levels, sleep disturbances, limitations in daily activities, and confidence when leaving home). Item response values ranging from 0 to 5 (0 = no impairment). The total score was obtained by summing the response values for each item (ranging from 0 to 40). Higher scores indicate a greater impact of the disease on the patient's health and life [35]. Within the CAT, 0–9 is considered a low impact, 10–20 is considered a moderate impact, 21–30 is considered a high impact, and 31–40 is considered a very high impact [36]. Pulmonary function test will be evaluated at weeks 0, 12 and the end of follow-up. Pulmonary Function Test is the gold standard for COPD diagnosis and severity grading. The main indices to be collected include: forced expiratory volume in one second (FEV1), FEV1%pred, forced vital capacity (FVC) and FEV1/FV [37]. The 6-MWD will be evaluated at 12 weeks of enrollment and every 3 months during the follow-up period. The 6MWT is a test commonly used to objectively assess functional exercise capacity in patients with moderate-to-severe pulmonary disease. In the test, patients are asked to walk along a 30-m corridor for as for as possible for 6 minutes [38]. The SF-36 is used to measure self-reported health-related quality of life (HRQOL). SF-36 consists of 36 main questions covering 8 aspects (physical functioning, role physical, bodily pain, general health, vitality, social functioning, role emotional, and mental health). The total score is 0–100, with higher scores representing better quality of life [39]. An acute exacerbation of COPD is defined as an acute worsening of respiratory symptoms (increased dyspnea, cough and sputum purulence) that results in additional therapy [37, 40]. A mild exacerbation refers to patients treated with short-acting bronchodilators (SABDs) plus antibiotics and/or oral glucocorticoids; a severe exacerbation refers to patients requiring hospitalization or emergency care. We will count the number of mild/severe acute exacerbations in the next year for all subjects after the end of treatment. The mMRC, CAT, SF-36 scales, and number of acute exacerbations will be evaluated at weeks 0, 4, 8, and 12 of enrollment and every 3 months during the follow-up period. Data will be collected on 6-MWD at weeks 0 and 12 of enrollment and every 6 months during follow-up. Pulmonary function tests will be performed at weeks 0 and 12 of enrollment and at the end of the 1-year follow-up.

### Safety assessment

Theoretically, the most serious adverse reaction during needling is pneumothorax, so we have developed a strict procedure during the trial, including the selection of different lengths of needles, strict direction and depth of acupuncture. During acupuncture, patients may experience adverse reactions such as needle sickness, subcutaneous hemorrhage, hematoma, severe pain, infection, and needle breakage. Adverse events (AEs) that occur throughout the study will be recorded and reported, and patients will be treated promptly and reasonably. The occurrence, symptoms, duration, severity, treatment measures and disappearance time of AEs will be recorded in detail. When adverse reactions occur, clinicians judge whether to discontinue the trial based on the condition and report severe cases to the Project Management Office of Chengdu University of Traditional Chinese Medicine and the Ethics Committee within 24 h.

### Sample size calculation

As reported in previous studies [15, 41], the mean improvement in SGRQ was -7.1 for patients in the three times a week acupuncture group, the mean change in SGRQ was -2 for patients in the SA group, the mean change in SGRQ was -0.8 for patients in the blank control group, with a standard deviation of 14.2. Using a two-tailed test with a category 1 error of 0.05 and a statistical efficacy of 80%, a sample size of 88 cases per group was calculated, with a maximum of 20% of subjects allowed to drop out or disengage for a required sample size of 110 cases per group. The once a week and twice a week acupuncture groups were set up with the same sample size as the three times a week acupuncture groups, and a total of 550 subjects were included.

### Statistical analysis

Analyses will be performed according to the intention-to-treat (ITT) principle and will be statistically analyzed for all randomized participants. Missing data will be filled in using the multiple imputation method. Statistical analysis will be performed by the Chinese Cochrane Center, West China Hospital, Sichuan University, China and SAS software will be used for statistical analysis. Quantitative indicators are described using mean, standard deviation, median, 25th percentile and 75th percentile. Categorical indicators are described using frequencies and percentages. The normality of the data will be verified by the Kolmogorov–Smirnov test (K-S test). Equality of variance between observed variables will be verified by Levene's Test. Baseline data will be analyzed using chi-square or Fisher's exact test for categorical indicators and ANOVA or the Kruskal–Wallis H test for quantitative indicators.

The primary outcome will be analyzed by generalized linear mixed effects model. Fixed effects included group, age, COPD sub-type, and years of smoking; random effects included sub-center, time, and time × group interaction. Secondary outcomes will be analyzed using linear mixed effects models, with fixed and mixed effects containing the same variables as the primary outcome indicators.

We will include participants who completed no less than 80% of their treatments in the per-protocol set (PPS). Several sensitivity analyses will be conducted to examine the robustness of the finding. First, we will exclude the participants with acute exacerbations during treatment period. Second, we will exclude the participants who have infected COVID-19 during treatment period. We will analyze each of the subgroups that may potentially influence the outcomes, including: 1) Age: < 60 or > 60; 2) Smoking history: yes or no; 3) FEV1%pre: ≤ 50 or > 50; 4) Type of COPD: chronic bronchitis type or emphysema type. Statistical test will be a 2-sided test, with *P* < 0.05 considered statistically significant.

### Treatment compliance

The number of expected and actual treatments for each patient will be counted to assess the patient's compliance. In patients whose treatment is discontinued due to an acute exacerbation, the patient will continue to complete the rest of the treatment after a week of stabilization. Patients who have been quarantined due to COVID-19 will continue to complete the rest of their treatment once they are unblocked. A patient will be regarded as compliant if receiving > 80% of the expected treatments.

### Quality control

The trial protocol was reviewed and revised by experts in acupuncture, respiratory medicine, methodology, and statistics. Standard operating procedures (SOPs) will be developed in advance, and training sessions will be held before the start of the study to provide standardized training for investigators at each center and to ensure that each researcher is familiar with the research process and the specific implementation details. The participants in the trial should be fixed, and the recording methods and judgment criteria should be unified to ensure that the scale assessment, data management, and completion of the CRF are carried out under uniform standards. The data inspector will conduct quality control checks at each research center every 3 months to ensure that all elements of the study protocol have been strictly adhered to, as well as the correctness of the completed information, and to generate quality control analysis reports.

### Data collection, management and monitoring

Data will be collected by telephone or on-site questioning by researchers not involved in the statistical analysis and treatment. The statistical company and the research team are jointly responsible for the data management of this project. The investigator fills out the clinical trial record requirements: timely, accurate, complete, standardized and truthful. The monitor verifies that the investigator follows the trial protocol during the experiment and regularly checks subjects' informed consent and inclusion at each sub-center. Data entry personnel will need to be trained to perform data entry. The CRF will be filled into the Electronic Data Capture (EDC) system by two independent data entry operators to ensure the accuracy of the data. Any changes to the data will be tracked in the EDC system.

## Discussion

As the third leading cause of death worldwide, COPD affects patients’ health and quality of life and imposes a substantial economic burden on society.

The GOLD guideline states that the primary goal of managing stable COPD is to relieve symptoms and reduce the risk of future acute exacerbations. The GOLD recognizes the SGRQ as the gold standard for evaluating COPD patients’ symptoms. SGRQ is a comprehensive disease-specific quality-of-life health questionnaire used in clinical trials with high validity, sensitivity, and reliability. The SGRQ is systematic, rigorous, detailed, and comprehensive and consists of three main sections: mobility, respiratory symptoms, and impact on daily life. The severity of COPD patients is closely related to their quality of life, and it has been found that the effectiveness of treatments and interventions for COPD patients can be reflected in their quality of life. The SGRQ is generalizable, and its scores have been validated repeatedly for different countries, races, ages, genders, and severities of respiratory diseases. The questionnaire covers the physical, emotional, and social aspects of the patient's life, correlates well with other clinical symptoms [42, 43], and is superior to other assessment scales [44, 45]. Pearson's analysis showed that the SGRQ, mMRC, and CAT had a good correlation (*P* < 0.05) and were consistent in the assessment of COPD, and all of them could be used to assess the degree of lung function impairment. The CAT scale is comprehensive, highly reliable, easy to understand, and time-consuming. The mMRC is also widely used in clinical practice because of its reproducibility, authenticity, and sensitivity. The 6-MWT is an objective sub-maximal exercise test. Because most of the daily activities of patients with COPD are at the sub-maximal level, the 6-MWD can reflect the functional compensatory capacity of patients to complete daily physical activities. The 6-MWD indicates the patient's functional compensatory capacity to perform daily physical activities. It can be used as an objective tool to assess the extrapulmonary effects of the disease on patients [38]. SF-36, as a universal measurement scale, can reflect the quality of life of patients with chronic diseases to a large extent. The pulmonary function test is a noninvasive tool for studying respiratory physiology, an objective indicator for determining airflow limitation with good reproducibility, and the gold standard for diagnosing COPD. It is vital for the diagnosis and differential diagnosis of COPD and evaluating severity, disease progression, prognosis, and response to treatment.

A clinically ideal placebo/sham acupuncture control should meet three basic principles: (1) placebo acupuncture has no or almost no specific therapeutic effect; (2) the needle is applied to a site that has no therapeutic effect; and (3) the subject cannot perceive the difference between placebo acupuncture and therapeutic acupuncture. However, due to the complexity of the acupuncture operation process, it is not easy to fully meet the above requirements. In most previous studies, non-acupoints or points on meridians not related to the treatment of disease were chosen as sham acupuncture points. One study found that acupoints have special efficacy in hand rheumatoid arthritis compared to non-acupoints. However, due to the nature of the disease and the size of the space of the acupoints, it is not possible to prove that the acupoints taken on the meridian side are non-acupoints [46]. Considering the above reasons, we will perform placebo acupuncture using flat-tipped needles at the same acupoints as the acupuncture group. In order to achieve a better blinding effect by convincing the patient, we will let the patient feel the pain of his/her stabbing but will not pierce the patient's skin. When inserted, the blunt tips of these special placebo needles produce a mild sting on the skin's surface but do not puncture the skin. Moreover, the needle retention on the base after needle insertion simulates regular needle insertion, which, combined with the retention and exit of the needle after needling, the subject can perceive it as an actual needling for optimal blinding. Considering that the natural course and regression of COPD disease are unpredictable, we additionally set up a blank control group that was not given any interventions except for regular medication.

This study has strict nadir criteria, and respiratory diseases that impact pulmonary function tests (for example, bronchial asthma, bronchiectasis, lung cancer, and lung resection surgery) will be excluded. Any additional use of Western medicine will be recorded in detail. Acupoint selection is a critical factor in acupuncture treatment. The acupoints selected for this study were based on data mining of modern acupuncture prescriptions for COPD. The most commonly used safe and effective acupoints were selected with consultation with clinical acupuncturists. The patient's prone position was also considered for easy manipulation [14, 47]. At the time of signing the informed consent form, subjects in the waiting-list group control group, the SA group, will be promised that compensatory treatment will be given at the end of the study. In this way, potential problems caused by patient disappointment will be overcome.

Inevitably, this study also has some limitations. First, due to the acupuncture operation's nature, it is impossible to blind the acupuncturist, which may also lead to bias. Second, the blunt-tipped needle touching the skin may have some stimulating effect on the acupoints, therefore it is necessary for us to set up a waiting-list control group.

## Conclusion

In conclusion, this study is a multicenter, randomized controlled trial to objectively evaluate the effectiveness and safety of acupuncture for stable COPD. It is the first RCT to investigate different acupuncture frequencies for stable COPD. It is proposed to explore the quantitative effect pattern of acupuncture from the perspective of acupuncture frequency influencing the acupuncture effect and to provide a reference for optimizing clinical decisions of acupuncture for chronic diseases.

### Trial status

The recruitment process began on April 17, 2022, and is anticipated to end on December 31, 2023.

### Supplementary Information

**Additional file 1.** SPIRIT 2013 Checklist: Recommended items to address in a clinical trial protocol and related documents*.

## Data Availability

Not applicable; no data have yet been generated.

## Abbreviations

COPD: Chronic obstructive pulmonary disease
CAT: COPD assessment test
CRF: Case report form
EDC: Electronic data capture
LFSI: Lung function screening indicator
MCID: Minimal clinically important difference
mMRC: Modified-medical research council
PPS: Per-protocol set
PFT: Pulmonary function test
RA: Research assistant
MA: Manual acupuncture
SF-36: Medical outcomes study short form-36
SGRQ: St. George’s respiratory questionnaire
SA: Sham acupuncture
6-MWT: Six-minute walk test
6-MWD: Six-minute walk distance
